# The collaborative study on the genetics of alcoholism: Brain function

**DOI:** 10.1111/gbb.12862

**Published:** 2023-08-17

**Authors:** Jacquelyn L. Meyers, Sarah J. Brislin, Chella Kamarajan, Martin H. Plawecki, David Chorlian, Andrey Anohkin, Samuel Kuperman, Alison Merikangas, Gayathri Pandey, Sivan Kinreich, Ashwini Pandey, Howard J. Edenberg, Kathleen K. Bucholz, COGA Collaborators, Laura Almasy, Bernice Porjesz

**Affiliations:** ^1^ Department of Psychiatry and Behavioral Sciences State University of New York Downstate Medical Center Brooklyn New York USA; ^2^ Department of Psychiatry, Robert Wood Johnson Medical School Rutgers University New Brunswick New Jersey USA; ^3^ Department of Psychiatry Indiana University Bloomington Indiana USA; ^4^ Department of Psychiatry Washington University in St. Louis St. Louis Missouri USA; ^5^ Department of Psychiatry University of Iowa Iowa City Indiana USA; ^6^ Department of Biomedical and Health Informatics Children's Hospital of Philadelphia Philadelphia Pennsylvania USA; ^7^ Penn‐CHOP Lifespan Brain Institute University of Pennsylvania Philadelphia Pennsylvania USA; ^8^ Department of Genetics, Perelman School of Medicine University of Pennsylvania Philadelphia Pennsylvania USA; ^9^ Department of Biochemistry and Molecular Biology Indiana University Bloomington Indiana USA

**Keywords:** alcohol use disorder, EEG, ERO, ERP, family, genomics, neurocognitive, neurodevelopment, neurophysiology, neuropsychological

## Abstract

Alcohol use disorder (AUD) and related health conditions result from a complex interaction of genetic, neural and environmental factors, with differential impacts across the lifespan. From its inception, the Collaborative Study on the Genetics of Alcoholism (COGA) has focused on the importance of brain function as it relates to the risk and consequences of alcohol use and AUD, through the examination of noninvasively recorded brain electrical activity and neuropsychological tests. COGA's sophisticated neurophysiological and neuropsychological measures, together with rich longitudinal, multi‐modal family data, have allowed us to disentangle brain‐related risk and resilience factors from the consequences of prolonged and heavy alcohol use in the context of genomic and social‐environmental influences over the lifespan. COGA has led the field in identifying genetic variation associated with brain functioning, which has advanced the understanding of how genomic risk affects AUD and related disorders. To date, the COGA study has amassed brain function data on over 9871 participants, 7837 with data at more than one time point, and with notable diversity in terms of age (from 7 to 97), gender (52% female), and self‐reported race and ethnicity (28% Black, 9% Hispanic). These data are available to the research community through several mechanisms, including directly through the NIAAA, through dbGAP, and in collaboration with COGA investigators. In this review, we provide an overview of COGA's data collection methods and specific brain function measures assessed, and showcase the utility, significance, and contributions these data have made to our understanding of AUD and related disorders, highlighting COGA research findings.

## Graphical abstract

In this review, we provide an overview of the neurophysiological and neuropsychological measures that have been collected within the COGA sample. We also provide illustrative examples of how these data, in combination with the rich genetic and phenotypic longitudinal data available, have advanced our understanding of the etiology and consequences of alcohol use disorder and related disorders.

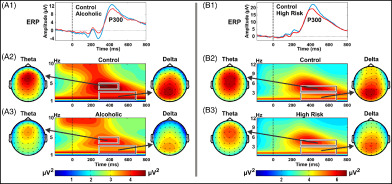

## 1 INTRODUCTION

Alcohol use disorder (AUD) and related health conditions result from a complex interaction of genetic, neural and environmental factors, with differential impacts across the lifespan.^1^, ^2^, ^3^, ^4^ The Collaborative Study on the Genetics of Alcoholism (COGA) is a multi‐site, interdisciplinary study that uses a family‐based research strategy focused on families densely affected with AUD and community comparison families. The goal of COGA is to elucidate the genetic and molecular mechanisms underlying the predisposition for AUD, uncovering neurobiological processes that potentially mediate genetic influences, and characterizing the interplay of genetic and environmental risk factors.^1^ Briefly, multi‐modal data has been collected on family members (aged 7–97), including clinical and behavioral measures derived from an age‐tailored version of the Semi Structured Assessment for the Genetics of Alcoholism (SSAGA; see 1. Overview), various questionnaires on behavior, personality and social functioning, blood samples for genetic analyses, and a neuropsychological and neurophysiological battery to assess brain function (see 2. Sample and Clinical Data). Data were collected at seven study sites across the United States, each lab operating under the same protocol, resulting in collection of similar data across all sites that could be pooled in analyses.^5^, ^6^, ^7^, ^8^ An overview of the overarching objectives of COGA, as well as detailed description of the COGA study data collection are provided elsewhere in this issue (see 1. Overview; 2. Sample and Clinical Data; 4. Genetics; 5. Functional Genomics).

From its inception, COGA has focused on the importance of brain function as it relates to the risk and consequences of alcohol use and AUD, through the examination of noninvasively recorded brain electrical activity and neuropsychological tests. A landmark study in 1984 from Begleiter and Porjesz reported that sons of fathers affected with AUD displayed neurophysiological differences even before they ever consumed alcohol.^9^ These findings, which were replicated in both males and females by several independent research groups throughout the world and within both male and female offspring of the original COGA participants,^10^, ^11^, ^12^, ^13^, ^14^, ^15^, ^16^, ^17^, ^18^ changed the field's thinking from an assumption that all neural anomalies were a result of prolonged alcohol consumption to the hypothesis that neural differences before onset of use may also contribute to risk for AUD. Decades later, COGA's sophisticated neurophysiological and neuropsychological measures, together with the rich multi‐modal family and longitudinal data collection, have allowed us to disentangle brain‐related risk and resilience factors from the consequences of prolonged and heavy alcohol use in the context of genomic, psychosocial and environmental influences over the lifespan.

Alcohol use disorder is a complex and multifactorial disorder, making the understanding of pathways from genetic predispositions to phenotypic expression of AUD a huge challenge. It was initially theorized that alcohol‐related measures of brain function such as neurophysiological markers, described as “endophenotypes” or “intermediate phenotypes,” may reflect simpler genetic processes and be more proximal to the genes involved in the predisposition to AUD.^19^, ^20^, ^21^ However, regarding AUD, research has found that brain‐based phenotypes are also complex, multi‐factorial traits that reflect the contribution of many genetic variants of small effect from across the genome. Nonetheless, measures of brain function associated with AUD, such as neurophysiological markers of risk, continue to be extremely useful in delineating underlying mechanisms (e.g., attention, impulsivity) associated with liability for complex conditions such as AUD. Over multiple decades, COGA has led the field in characterizing genetic variation associated with brain functioning and has advanced understanding of how genomic risk affects the development and course of AUD.

To date, the COGA study has amassed brain function data on over 9871 participants, including 7837 with data at more than one time point (Table 1). Please see *2. Sample and Clinical Data* for an overview of the different data collection waves, the research question guiding the focus of the data collection, the numbers of participants assessed at each, and tabular summaries of their characteristics. Briefly, data collection has focused on different participant subsets over time, depending on the research emphasis. Table 1 presents a comparison of key characteristics across the different “waves” of data collection. For several different subsets of participants and data collections, there are multiple assessments available on the same individuals. However, only the “Prospective study” which focused on assessments of youth aged 12–22 at two‐year intervals was specifically designed as a longitudinal study. 85% of this subsample has at least three follow‐up assessments, and a “non‐response analysis” indicated that individuals who did not return for follow‐ups were younger, but no significant differences regarding other key characteristics have been observed. We continue to take measures to ensure minimal impact of “missing data” on our study's findings. There is notable diversity in age (ranging from 7 to 97), gender (52% female), self‐reported race and ethnicity (28% Black, 9% Hispanic). These data are available to the research community through several mechanisms, including directly through the NIAAA, through dbGAP, and in collaborations (see 1. Overview). In this review, we detail COGA's data collection methods for the specific brain function measures assessed, showcase their utility, significance and contributions to our understanding of AUD and related disorders, and highlight COGA research findings.

**TABLE 1:** COGA Brain function subsample description (as of January 2023).

COGA Studies	All	Initial study	Prospective study	Lifespan study (ongoing)	
Collection years	1989–2023	1989–2004	2004–2019	2019–2023 **data updated 1/2023	
Life stage (age range)	Lifespan (7–97)	Lifespan (7–79)	Adolescence and young adulthood (12–32)	Midlife (33–49)**^prospective study participants^	Later Life (50+)**^initial study participants^
Sample sizes	9871	8098	2979	870	1335
Longitudinal subsamples					
1+ assessments	7837	7449	2794	870	1083
2+ assessments	644	200	2412	851	1255
3+ assessments	505	–	1997	799	10
4+ assessments	470	–	1486	745	1
Genomic Data Available	9076	6204	2872	835	1299
*Key characteristics*					
Age range at baseline (mean)	7–79 (38.9)	7–79 (39.8)	12–32 (16.9)	33–41 (36.5)	50–97 (65.5)
Female	51.9%	51.2%	51.5%	57.0%	60.4%
Black (self‐identified)	27.9%	18.7%	29.0%	21.8%	16.6%
Hispanic (self‐identified)	8.9%	6.0%	11.8%	11.7%	5.9%
DSM‐IV alcohol dependence	24.3%	31.0%	17.5%	24.4%	41.8%
DSM‐5 alcohol use disorder	47.5%	53.5%	41.4%	52.0%	62.5%

*Note*: Description of individuals within COGA that have neurophysiological and neuropsychological data. A full list and description of all measures can be found in the Supplemental Methods. A description of the full COGA sample can be found in 2. Sample and Clinical Data.

## 2 COGA'S MEASURES OF BRAIN FUNCTION

In this section, we provide a brief description of COGA's neurophysiological and neuropsychological assessment battery and highlight the unique information that each metric can offer to improve our understanding of the role of brain function in AUD. Tables 2 and 3 provide an overview of selected measures and findings from COGA. Detailed information about data collection and processing can be found in the Supplemental Material.

**TABLE 2:** Overview of neurophysiological and neuropsychological testing.

COGA Studies	All	Initial Study	Prospective Study	Lifespan study (ongoing)	
Collection years	1989–2023	1989–2004	2004–2019	2019–2023**data updated 1/2023	
Life stage (age range)	Lifespan (7–97)	Lifespan (7–79)	Adolescence and young adulthood (12–32)	Midlife (33–49)**prospective study participants	Later Life (50+)**initial study participants
Neurophysiological experiments					
Resting‐state EEG^a^ ^,^ ^b^ ^,^ ^c^ ^,^ ^d^	✓	✓	✓	✓	✓
Visual oddball task^a^ ^,^ ^b^ ^,^ ^c^ ^,^ ^d^	✓	✓	✓	✓	✓
Auditory oddball^a^ ^,^ ^b^ ^,^ ^c^ ^,^ ^d^	✓	✓	✓	✓	✓
Semantic priming^a^ ^,^ ^b^ ^,^ ^c^ ^,^ ^d^	✓	✓	✓	✓	✓
Go‐NoGo task^b^ ^,^ ^c^			✓	✓	
Monetary gambling task^b^ ^,^ ^c^			✓	✓	
Continuous performance test^b^ ^,^ ^c^			✓	✓	
Color/word stroop^b^ ^,^ ^c^			✓	✓	
Auditory novel stimuli^b^ ^,^ ^c^			✓	✓	
Cognitive/affective stroop^b^ ^,^ ^c^ ^,^ ^d^			✓	✓	✓
Mismatch negativity^a^		✓			✓
Bereitschafts potentials^a^		✓			
Contingent negative variation^a^		✓			
Object recognition^a^		✓			
Intertrial interference^a^		✓			
Neuropsychological tasks					
Tower of London^b^ ^,^ ^c^			✓	✓	
Visual span test^b^ ^,^ ^c^			✓	✓	
NIH Toolbox cognitive battery^c^ ^,^ ^d^				✓	✓
NIH Toolbox emotional battery^c^ ^,^ ^d^				✓	✓
Porteus maze test^a^ ^,^ ^d^		✓			✓
TRAILS A&B^a^ ^,^ ^d^		✓			✓
Wechsler Adult & Child Intelligence Scales‐revised^a^ ^,^ ^d^		✓			✓
California verbal learning test adult and child^a^ ^,^ ^d^		✓			✓
Ravens progressive matrices^a^ ^,^ ^d^		✓			✓
Wide range achievement test revised^a^ ^,^ ^d^		✓			✓

*Note*: A full list and description of all measures can be found in the Supplemental Methods. A description of the full COGA sample is provided in: 2. Sample and Clinical Data.

^a^ Initial COGA study (1989–2004).

^b^ Prospective Study Sample: Multiple assessments every 2 years during adolescence and young adulthood from 2004 to 2019. Added Frontal Lobe Battery in Prospective study to assess aspects of frontal lobe in development during that period. Phenotypes derived from this data can be used for studies of neurodevelopmental trajectories.

^c^ Lifespan Project: Midlife (ML): Prospective Study sample as they enter early mid‐life/midlife (33–49);

^d^ Lifespan Project Latelife (LL): Initial sample now aged >50 (One or two previous assessments ~20 years ago).

**TABLE 3:** Selected COGA Neurophysiological and Neuropsychological Findings.

Task	Key Phenotypes: Findings and Significance
Resting EEG^a^ ^,^ ^b^ ^,^ ^c^ ^,^ ^d^	*Theta power* (3–7 Hz): *Phenotypic findings*: Increased resting state posterior theta power observed among individuals with AUD, but not high‐risk offspring, a pattern similar to what has been observed in older individuals with dementia, suggesting deficient information processing capacity.^22^ *Beta power* (13–28 Hz): *Phenotypic findings*: Increased resting beta power observed in both individuals with AUD^23^ and in their high‐risk offspring,^24^ suggesting that it antecedes alcohol misuse, and may be a neural marker of vulnerability; Suggested that increased beta power in resting state may be an electro‐physiological index of an imbalance in excitation‐inhibition. *Genetic findings*: Highly heritable; Linkage was observed between beta power and a GABA_A_ receptor cluster, and association was observed with *GABRA2*,^25^ AUD, other substances, and related disorders including “precursor” externalizing phenotypes. In GWAS studies, *fast* beta power was associated with *DSE, ZEB2 and CTBP2*, with the latter two genes also associated with AUD.^26^ Beta power associated with *BCHE* in families of African ancestry, as well as AUD in adults and heavy drinking in offspring.^27^ *Inter/Intrahemispheric EEG Coherence*: *Phenotypic findings*: Increased interhemispheric coherence observed among individuals with AUD and among high‐risk offspring.^19^, ^28^ *Genetic findings*: EEG coherence measures were found to be heritable using family data; Increased theta interhemispheric coherence observed among individuals with AUD^19^, ^28^ was associated with *CHRM2* and *GABRA2*. GWAS of theta coherence identified loci on chromosome 18 associated with myelin regulation, severe alcohol use and corpus callosum volume.^29^ Polygenic scores (PGS) for AUD influenced neurodevelopmental trajectories of alpha inter‐hemispheric coherence in males, with increased risk for AUD, opioid use disorder, poorer planning, problem solving and visuospatial working memory. PGS for neuropsychiatric disorders have age‐ and sex‐specific influences on high alpha connectivity (e.g., schizophrenia affects high alpha connectivity among males between aged 12–16).^30^ *Functional Connectivity* (eLORETA lagged connectivity) *Phenotypic findings*: Dysregulation in network communication in Default Mode Network (DMN) seen in those who developed alcohol‐related memory problems 20 year later.^31^ *Genetic findings*: PGS for AUD, along with other multimodal features (including functional connectivity in default mode network), are associated with alcohol‐related memory problems.^31^
*Visual oddball task* (VPc)^a^ ^,^ ^b^ ^,^ ^c^ ^,^ ^d^	*ERP: P300 amplitude to targets* (Pz): *Phenotypic findings*: Low P300 observed among individuals with AUD and high‐risk offspring^32^; parenting (i.e., closeness with father) was associated with increased P3; suggests that parental closeness may promote brain function, and resilience against development of AUD in high‐risk offspring.^33^ *Genetic Findings*: Significant heritabilities for P300 using family data; P300 associated with *CRHR1* and AUD.^34^ *ERO: Frontal Theta to targets*: *Phenotypic findings*: Low frontal theta ERO observed among individuals with AUD and high‐risk offspring^35^, ^36^; associated with impulsivity and externalizing traits^37^, ^38^, ^39^; Parenting (i.e., closeness with father) was associated with higher frontal theta power; parental closeness promotes healthy brain function and resilience against the development of AUD in high‐risk offspring.^33^ *Genetic Findings*: Frontal theta ERO associated with *CHRM2* and *GRM8* (in linkage studies),^40^, ^41^ *KCNJ6* and *HTR7* (with GWAS studies)^42^, ^43^; age and sex specific influences are observed in the association of *KCNJ6* variants and theta ERO trajectories.^44^ *ERO: Posterior Delta to targets*: *Phenotypic findings*: Low posterior delta observed among individuals with AUD and high‐risk offspring.^35^, ^36^ *Genetic Findings*: Discrete Time Survival Analysis (12–25 years, COGA)^45^; Low delta predicted early onset regular alcohol use.^45^ *ERO: Gamma to targets* *Phenotypic findings*: Gamma differences observed among individuals with AUD and high‐risk offspring.^46^, ^47^
Auditory oddball task (AOD)^a^ ^,^ ^b^ ^,^ ^c^ ^,^ ^d^	*ERP: P300 amplitude to targets*: *Phenotypic findings*: Decreased auditory processing was observed among individuals with AUD and high‐risk offspring.^48^, ^49^, ^50^, ^51^ *ERO: Theta to targets*: *Phenotypic findings*: Decreased auditory processing was observed among individuals with AUD and high‐risk offspring.^48^, ^49^, ^50^, ^51^ *Genetic Findings*: Age and sex specific influences are observed in the association of KCNJ6 variants and theta ERO trajectories during auditory task.^44^, ^52^
Semantic priming task (ANT)^a^ ^,^ ^b^ ^,^ ^c^ ^,^ ^d^	*ERP: N400 amplitude to primed and unprimed words* *Phenotypic findings*: Among individuals without AUD, primed words (preceded by antonym) have faster processing speed and need less processing than unprimed words (preceded by unrelated words) than individuals with AUD; Individuals with AUD and high‐risk offspring have the same N400 to primed and unprimed words suggesting reduced flexibility in cognitive networks and a lack of resource optimization.^53^, ^54^
Go/NoGo task (GNG)^b^ ^,^ ^c^	*ERP: P300 and N200 amplitude during ‘Go’ and ‘NoGo’* *Phenotypic findings*: Lower N200/P300 amplitude and current density during “Go” and “NoGo” condition among individuals with AUD and high‐risk offspring, suggesting impaired response inhibition and execution.^55^, ^56^, ^57^ *ERO: Frontal Theta during ‘NoGo’*: *Phenotypic findings*: Lower frontal theta ERO during “NoGo” among individuals with AUD,^58^, ^59^, ^60^ high‐risk offspring and adolescents and young adults exposed to childhood sexual trauma suggesting frontal lobe dysfunction during response inhibition.^61^ Childhood sexual trauma influenced developmental trajectories of frontal theta power during ‘NoGo’, associated with increased risk for AUD, MDD and PTSD.^61^
Monetary Gambling Task (MGT)^b^ ^,^ ^c^	*ERP: P300 and N200 amplitude* *Phenotypic findings*: Low P300 and N200 during reward processing was observed among individuals with AUD^62^ and high‐risk offspring.^63^ *ERO: Frontal Theta* *Phenotypic findings*: Low theta ERO activation in frontal regions during evaluation of loss observed among individuals with AUD^37^ and high‐risk offspring,^64^ suggesting frontal cortical and subcortical deficits in reward networks; increased EXT; and impulsivity, that negatively correlated with frontal theta ERO to loss, and risky behavior.^37^, ^64^ *Genetic findings*: ERO theta during reward processing was associated by *KCNJ6 SNP* rs702859.^65^
CATs Tower of London Test (TOLT)^b^ ^,^ ^c^	*Phenotypic findings*: Planning and problem‐solving (frontal executive function), were less efficient in high‐risk offspring, particularly those who experienced early trauma^66^, ^67^; Females both exposed to sexual assaultive trauma, and from families more densely affected with AUD, displayed higher rates of PTSD symptoms. Young adults with exposure to non‐sexual assaultive trauma had less efficient neurocognitive performance (planning and problem solving skills), possibly suggesting frontal lobe deficits.^66^, ^67^ *Genetic findings*: Individuals with higher AUD PGS demonstrated deficits in neuropsychological performance, including poorer planning and problem solving skills on the Tower of London task.^29^
CATs Visual Span Test (VST)^b^ ^,^ ^c^	*Phenotypic findings*: Visuospatial memory span and working memory deficiency in high‐risk offspring.^68^ *Genetic findings*: Individuals with higher AUD PGS demonstrated deficits in visuospatial memory and working memory on the visual span test.^29^

^a^ Initial COGA Sample (1989–2004).

^b^ Prospective Study Sample: Multiple assessments every 2 years during adolescence and young adulthood from 2004 to 2019. Added Frontal Lobe Battery in Prospective study to assess aspects of frontal lobe in development during that period. Phenotypes derived from this data can be used for studies of neurodevelopmental trajectories.

^c^ Lifespan Project: Midlife (ML): Prospective Study sample as they enter mid‐life (33–49).

^d^ Lifespan Project Latelife (LL): Initial sample now aged >50 (One or two previous assessments ~20 years ago).

### 2.1 Neurophysiological assessments

**FIGURE 1:**
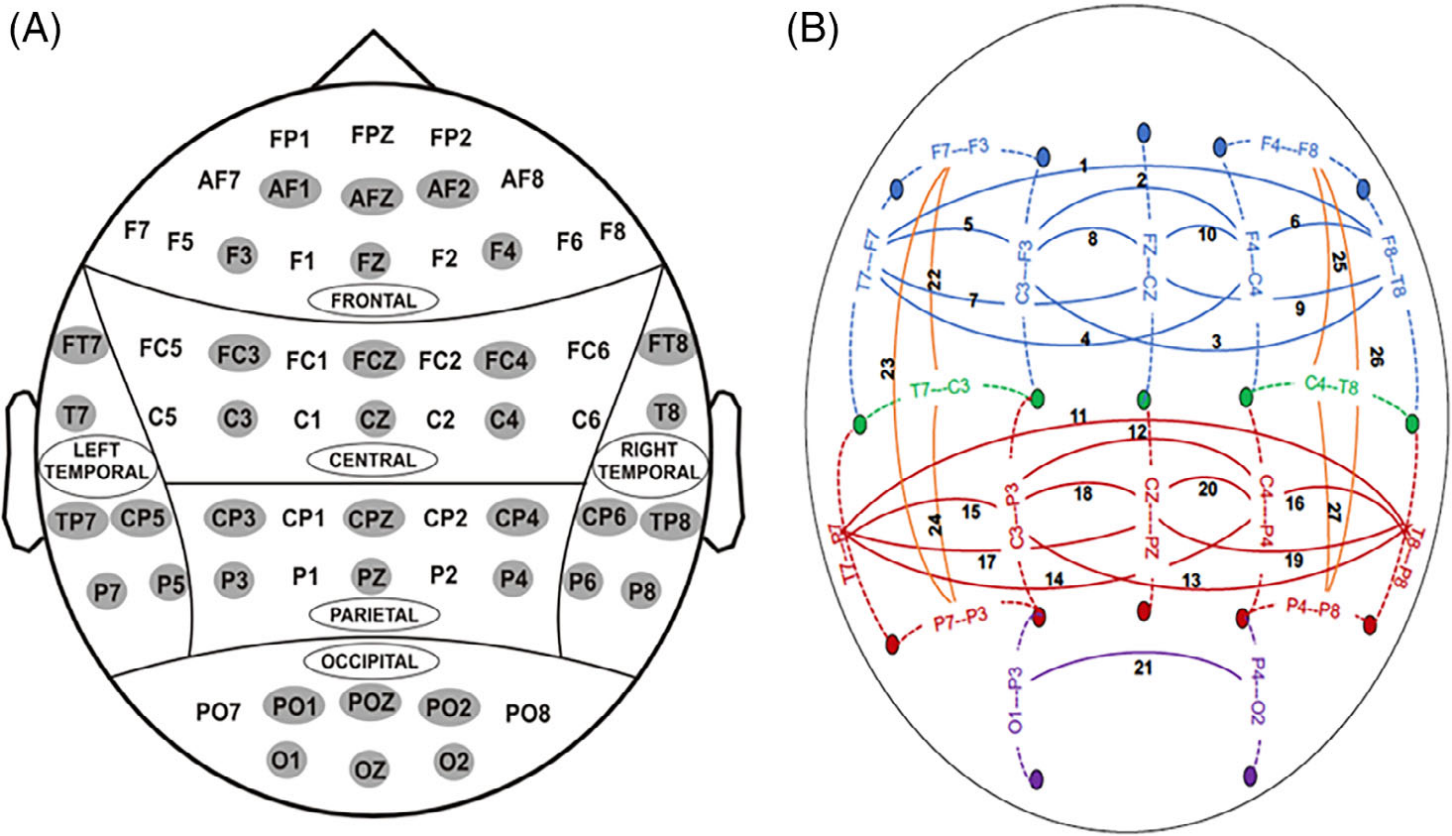
EEG Electrodes and Coherence Pairs used for Electrophysiological Recordings and Analyses in COGA. (A) Sixty‐one scalp electrodes are used to record EEG and ERPs in frontal (F), central (C), parietal (P), occipital (O), left‐temporal (LT) and right‐temporal (RT) regions. Even numbers signify the right side of the head, while odd numbers signify the left side, and Z signifies the center; the numbers indicate coordinates ascending from the center to the periphery of the scalp. (B) Bipolar electrode pairs used as the coherence measures of resting state EEG.

Using electroencephalography (EEG) techniques, COGA's neurophysiological battery records voltage oscillations originating from the cortical surface of the brain, by use of non‐invasive scalp electrodes (Figure 1A). These high‐temporal resolution recordings provide millisecond by millisecond indices of ensembles of neurons firing in synchrony during resting state (resting EEG) and during sensory, behavioral and cognitive tasks, from which event‐related potentials (ERPs) and event‐related oscillations (EROs) are derived. Although fMRI provides superior spatial (including subcortical) resolution to precisely pinpoint brain structures involved, the fine time‐scale and wide range of frequency bands provided by neurophysiological recordings may prove important for understanding subtle neural communication during sensory and cognitive processing relevant to neuropsychiatric outcomes. COGA's neurophysiological battery is designed to assess aspects of brain function that may be aberrant in AUD and/or involved in a vulnerability to increase risk to develop AUD. COGA's neurophysiological and neuropsychological batteries have evolved over time, both to stay current with state‐of‐the‐science development of brain function measures, and to the best capture brain functioning throughout the different stages of the lifespan focused on with each phase of data collection (e.g., adolescence and young adulthood, later‐life). Table 2 provides an overview of the specific assessments at each phase of data collection and Table 3 for an overview of selected measures and significant findings in COGA).

#### 2.1.1 Resting state EEG

During “resting state,” defined here as when one is not engaged in a cognitive task, the brain still emits spontaneous, rhythmic activity. Resting EEG measures a complex signal of voltage oscillations comprising a wide range of spectral frequencies (i.e., number of waves per second, Hz), that are subdivided into bands: delta (1–3.5 Hz), theta (3.5–7.5 Hz), alpha (7.5–12.5), beta (12.5–28) and gamma (above 28 Hz). The resting EEG is stable and highly heritable across all frequency bands.^69^ These EEG rhythms are indicators of global brain states from the alert‐awake state to drowsiness and stages of sleep. In healthy adults, alpha and beta frequencies predominate the awake resting EEG, with alpha rhythm dominating during relaxation parietal‐occipitally and beta seen throughout the scalp with mental activation.^70^ In COGA, resting‐state EEG is recorded while participants have their eyes closed (4.25 min) and again while they have their eyes open (4.25 min). Measures of resting EEG analyzed in COGA are (1) *EEG power* (absolute and relative) in each frequency band, representing the amount of neural activity in these frequency bands,^71^ and (2) metrics of *EEG functional connectivity* (coherence and source connectivity (eLORETA) that reflect the degree to which different brain regions/neural networks^71^ communicate with each other.

#### 2.1.2 Time‐locked EEG during cognitive tasks (ERPs, EROs)

**FIGURE 2:**
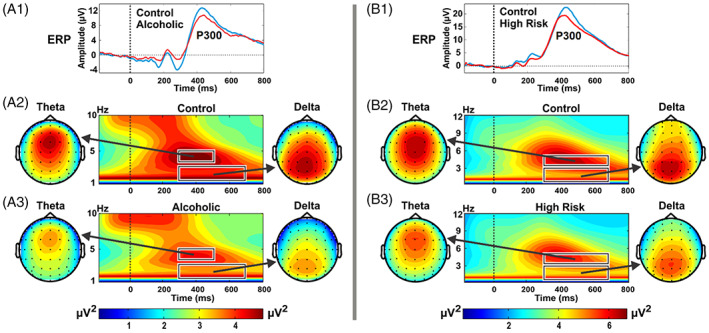
Brain wave characteristics in individuals with AUD (left panels, A1–A3) and their children, who are at high risk to develop AUD, due in part to genetic influences through family history (right panels, B1–B3) during a mental task called a “visual oddball task” in which they were asked to press a button only to a specific “target” image on a computer screen, while ignoring other more frequent images. Brain responses only to the target stimulus are illustrated in this figure. In A1 and B1 (top panels) you can see that both individuals with AUD^35^, ^77^ (red lines, left panel) and their high‐risk children^36^ from AUD families (red line, right panel) had smaller P3 or P300 waves (the large peak in top panel occurring between 300 and 700 ms after the target) compared to those unaffected (blue lines, left panel) and at low risk from community comparison families (blue lines, right panel). In A2 and A3, and B2 and B3 (middle and bottom panels) you can see the lower magnitude of brain waves oscillating at theta (4–7 Hz) and delta (1–3 Hz) frequencies during the P300 response in both individuals with AUD (left panel) and their high‐risk children (right panel) compared to those unaffected (left panel) and offspring from comparison community families (right panel); note that theta oscillations have a frontal focus while delta oscillations have a more posterior focus. These differences in brain wave characteristics between individuals affected compared to unaffected with AUD, and between offspring from AUD families compared to offspring from community comparison families indicate less activation and/or weaker synchronization of neural activity during this cognitive task in those with AUD and their high‐risk children.

In contrast to resting state EEG recordings, COGA also records EEG when participants are engaged in cognitive/behavioral tasks. Studies focusing on neural responses to discrete stimuli or in relation to behavioral responses typically measure ERP responses and EROs. ERPs reflect changes in voltage across time following the presentation of a stimulus or the emission of a response, relative to a pre‐stimulus baseline in the time domain.^72^ These small voltage fluctuations are difficult to see in a single trial, so by averaging this activity across trials, Activity that is time‐locked in relation to the event of interest summates while activity unrelated to the stimulus cancels out. ERP response is quantified in terms of positive‐ and negative‐going peaks or ‘components’ in the average signal waveform within particular time windows. Positive and negative ERP components are designated “P” and “N” respectively and numbered to reflect the approximate timing in milliseconds, or latency, of their peak (e.g., P300, N400).^72^ Earlier ERP components are theorized to reflect sensory and more ‘automatic’ processes related to perceptual registration of an event, whereas later components are theorized to reflect cognitive processing of events.^73^, ^74^ ERPs are interpreted based on mode of delivery (auditory, visual), frequency (frequent, rare) and content of the eliciting stimuli. The amplitude (measured as height of an ERP peak or trough, in microvolts) indicates the magnitude of neural resources that contributed to process a stimulus or event, whereas the latency (time of peak occurrence) reflects neural processing time.^75^ One of the most widely used ERP components in neuropsychiatric research is the P3 or P300, a large positive component, maximal at centroparietal electrodes and occurring between 300 and 700 ms after the stimulus onset^76^ (Figure 2). The P300 is related to the “significance” of a stimulus in a task, and not its physical features, reflecting attentional allocation and context updating processes in working memory.^78^, ^79^, ^80^, ^81^

Event‐related oscillations are time–frequency measures of superimposed EEG activity at different frequency bands that are temporally related to sensory and cognitive processing that contribute to ERPs^82^ (Further details can be found in the Supplementary Material and Figure 2). We can analyze these brain signals in terms of their time‐frequency components arising from various brain regions during sensory and cognitive tasks.^83^ While EROs are partitioned in the same frequency bands as resting EEG (e.g., delta, theta, alpha, beta, gamma), they are functionally different from spontaneous rhythms, with each frequency band underlying specific cognitive processes, namely, delta: signal detection, decision making; theta: conscious awareness, recognition memory; alpha: attentional resources; beta and gamma: sensory, integrative processes. Faster frequency oscillations (gamma and beta) are involved in shorter range local neural communication while slower frequencies (delta, theta and alpha) are involved in longer range neural communication in the brain.^84^ P300 responses are primarily the outcome of theta and delta EROs elicited during cognitive processing.^35^, ^82^ Theta oscillations peak earlier during the attentional and memory‐related aspects of the task and have a frontal focus, representing fronto‐limbic or cortico‐hippocampal interactions.^19^, ^77^, ^85^, ^86^ Delta oscillations peak at posterior regions and are involved with decision‐making and response selection aspects of the task and represent cortico‐cortical interactions.^19^, ^77^, ^86^, ^87^

#### 2.1.3 Neuropsychological assessments

COGA has also assessed various aspects of cognitive function with neuropsychological batteries since its inception, with emphasis on different aspects of cognitive function depending on the life stage of the sample under study (Table 2 details specific neurocognitive assessments at different waves of COGA). In the initial COGA study, a comprehensive, wide range of neuropsychological assessments were obtained on the sample (aged 7–79) to assess child and adult verbal, non‐verbal and fluid intelligence, abstract reasoning, planning, verbal learning and memory, reading, comprehensive and mathematical abilities, and visual attention. These tests include: Wechsler Adult Intelligence Scale Revised (WAIS‐R),^88^ Wechsler Intelligence Scale for Children Revised (WISC‐R),^89^ Wide Range Achievement Test Revised (WRAT‐R),^90^ Trail Making Test (TMT),^91^ Porteus Maze Test (PMT),^92^ and California Verbal Learning Test, Adult (CVLT‐A)^93^ and Child (CVLT‐C).^94^ During the subsequent waves of data collection (i.e., the prospective study of adolescents and young adults), we focused on neuropsychological assessments of frontal lobe function, which are undergoing maturation during adolescence and young adulthood. Neuropsychological assessments of frontal executive function, such as planning and problem‐solving abilities (Tower of London Task) and visuospatial working memory (Visual Span Test) from the Colorado Assessment Tests (CATs)^95^ were implemented as part of the frontal lobe focused battery of neurophysiological and neuropsychological assessments. These assessments of frontal lobe executive function were repeated during the current wave of data collection (i.e., the Lifespan Study) in the prospective sample as they entered mid‐life (33–41 years old). In the ongoing Lifespan Study, the NIH Toolbox Cognitive battery (NIHT‐CB)^96^ has been implemented to comprehensively assesses neurocognitive functions that are essential for effective daily life including, attention, processing speed, language, episodic memory, working memory and basic executive functions (inhibitory and attentional control, unconscious set‐shifting and maintenance aspect of cognitive flexibility). We have also implemented the NIH Toolbox Emotion Battery (NIHT‐EB)^97^ to assesses negative affect (anger, fear and sadness), psychological well‐being (general life satisfaction, meaning and purpose, and positive affect), social relationships (friendship, loneliness, positive peer interaction, social withdrawal, empathic behavior, peer rejection, perceived hostility, perceived rejection, emotional support, and instrumental support) and stress and self‐efficacy (perceived stress, self‐efficacy).

## 3 HOW DO NEURAL SIGNATURES ASSOCIATED WITH AUD HELP ELUCIDATE THE ROLE OF BRAIN FUNCTION IN THE RISK AND CONSEQUENCES OF ALCOHOL USE AND AUD ACROSS THE LIFESPAN?

From its inception, COGA has recognized the importance of individual differences in brain function as both an antecedent and consequence of AUD. We have implemented a comprehensive battery of neurophysiological measures designed to assess activation and communication in neural networks in resting state and neural processing during cognitive tasks found to be affected in AUD (e.g., attention, response inhibition, reward processing). With this approach, we have identified neurophysiological measures that are associated with AUD as well as neurophysiological measures that differ based on family history of AUD and are present in some family members, even before alcohol exposure. The neurophysiological and neuropsychological COGA data have been instrumental in demonstrating that variations in brain function are both antecedents to, and consequences of, the effects of prolonged and heavy alcohol consumption and AUD (Table 3 provides specific results with neural measures during resting state and cognitive tasks and measures of neurocognitive function). COGA data have been particularly useful in studying the role of brain function as a neural liability for risk of AUD, and determining which of the measures of brain function have utility in genetic studies of AUD. As indicated in Section 2 above, EEG phenotypes are highly heritable, and several have been found to be aberrant in densely affected AUD families, including their offspring, compared to the comparison families, including related to severity and earlier age of onset.

Since its earliest studies, COGA has found that low P300 amplitude, associated with AUD is also found in high‐risk offspring.^11^ The initial studies in COGA reported lower P300 amplitudes to target stimuli in both visual and auditory oddball tasks in those with AUD and their offspring, albeit more consistently in visual tasks.^77^ COGA have demonstrated that low parietal P300 to visual targets is more prevalent in members of dense AUD families, including their offspring compared to members of community comparison families.^11^, ^98^ More recently, COGA has demonstrated that higher family history density of AUD was associated with lower amplitude parietal P300, higher likelihood of AUD, and earlier onset of regular drinking.^32^ Thus, the longitudinal study of family members with multimodal measures can elucidate the important relationship between neural measures and family density of AUD in affecting risk of developing AUD as well as allow for examining how social environment may moderate risk factors (further detail provided in Section 3.3). COGA was a pioneer in developing and implementing ERO methods to understand neural mechanisms underlying P300 and AUD. Investigating the EROs in the visual oddball task, our investigators were the first to demonstrate that the low amplitude P300 in AUD was due to lower activation of frontal theta EROs and posterior delta EROs.^35^ Subsequently, COGA was the first to demonstrate that lower theta and delta EROs underlying P300 in the same task were more sensitive than P3 in discriminating between high‐risk and low‐risk offspring.^36^ Thus, reduced activation of P300 and the associated frontal theta and posterior delta ERO measures of brain function during the oddball task are indicative of problems with attention, memory and decision‐making processes, preceding the development of AUD. These visual oddball task measures are obtained in all COGA participants, from its inception through the current Lifespan project and are considered to be the “core” phenotypes that can be used for analyses on the whole sample as well as in longitudinal analyses (>7000 participants have data at more than one timepoint). Examining resting state EEG, COGA have reported increased EEG beta power (12–28 Hz) in individuals with AUD and in their offspring.^23^, ^24^ Beta rhythm is an index of neural excitability,^99^ and the increased beta power in individuals with AUD and at‐risk family members is indicative of an imbalance in excitation/inhibition in neural networks. While increased beta was found for both genders, it was more pronounced in males than females, and related to family density of AUD, especially increased in those with two or more first‐degree relatives with AUD.^77^ COGA has also found increased resting state EEG interhemispheric coherence (Figure 1B) in several frequency bands in individuals with AUD and their offspring; it was not found to be related to length of abstinence from alcohol.^19^, ^28^

When COGA launched its prospective study of adolescent and young adult offspring (2004–2019), a neurocognitive battery was included to assess frontal lobe function during this critical developmental time period (e.g., to examine executive control and reward networks). In a Go/NoGo task, individuals with AUD and their high‐risk offspring showed lower frontal N200, P300 and frontal theta ERO during response inhibition, indicating less activation in frontal areas.^55^, ^56^, ^57^, ^58^, ^59^, ^60^ A series of studies using a Gambling task reported low P300 amplitudes and less theta ERO activation in frontal regions during the loss condition in individuals with AUD and high‐risk offspring, indicating deficits in reward processing.^37^, ^62^, ^63^, ^64^ Taken together, these findings indicate frontal lobe deficits in executive control and reward networks in adolescents and young adults at risk for developing AUD.^58^, ^64^

In sum, resting state EEG, ERP and ERO phenotypes have been central to COGA's success in addressing the role of brain function and genomic risk in AUD. In the sections that follow, we highlight studies where these neurophysiological markers of risk have been instrumental in advancing understanding of how genomic risk affects AUD development and resilience across the lifespan.

### 3.1 How do genomic factors influence brain functioning across the lifespan and contribute to antecedents and resilience for AUD?

From its inception, COGA has successfully used heritable, reliable, quantitative measures of brain function, to identify key genes that are significantly associated with these neural endophenotypes*,* advancing our approaches as newer genomic research, technologies and methods emerge (see 2. Sample and Clinical Data). COGA is in a unique position to use its considerable multimodal data to contribute to, as well as move beyond GWAS. COGA's multidisciplinary research team has facilitated translation from genetic variants identified by GWAS to their influence on the developmental trajectories of EEG phenotypes and relationships to substance use and clinical outcomes in longitudinal studies, as well as to perform molecular and functional genetic studies to enhance understanding (see 4. Genetics and 5. Functional Genomics).

#### 3.1.1 Early linkage and association studies of neurophysiological (endo)phenotypes

Our initial genetic studies took advantage of COGA's family history of AUD density and sensitive neurophysiological measures to perform linkage and association studies. We first reported significant linkage with resting EEG beta power and a GABA_A_ receptor gene involved in inhibitory neural networks,^25^ that was subsequently found to be associated with SNPs in *GABRA2* and later with alcohol dependence, substance dependence, and related disorders, including precursor externalizing phenotypes.^100^, ^101^, ^102^ These findings suggest an imbalance in excitation‐inhibition (hyperexcitability, disinhibition) in family members at risk for AUD. We identified several other genes in our COGA linkage families using neurophysiological phenotypes that were indices of risk for AUD. We found linkage and association between theta EROs to targets in the visual oddball task and SNPs in *CHRM2* (cholinergic muscarinic receptor gene)^40^, ^103^ and *GRM8* (metabotropic glutamate receptor gene)^41^ that were also found to be associated with alcohol dependence and related phenotypes (e.g., depression^104^). These early genetic findings have been replicated and extended in several other samples throughout the world.^105^, ^106^, ^107^, ^108^, ^109^, ^110^, ^111^, ^112^, ^113^, ^114^ For example, in an independent sample of high‐risk offspring from multiplex AUD families, the association of variants within *CHRM2* and P300 amplitude was confirmed, particularly trajectories of P300 in young male offspring aged 8–12.^115^ Further, variation in *GABRA2* (and its interaction with *BDNF*) was associated with gray matter volumes, suggesting that inherited variation in these genes may promote early developmental differences in neuronal proliferation of the cerebellum in these high‐risk offspring.^116^ *GABRA2* was also implicated in a large meta‐analytic genetic association study of EEG beta power and remained significant independent of COGA data.^117^ In an independent sample, *GRM8* variants were also found to be associated with P300 amplitude during response inhibition, as well as with AUD and related disorders, with data suggesting that the association of *GRM8* and AUD may be mediated through an inherited instability in brain function that affects cognitive control.^118^ These early studies are still being evaluated in much larger GWAS studies of alcohol dependence and related phenotypes. We also found that increased interhemispheric resting state EEG coherence in AUD families, suggesting dysfunctional thalamo‐cortical and cortico‐cortical connectivity.^28^ This measure of coherence was significantly associated with SNPs in *GABRA2* at parieto‐occipital regions and SNPs in *CHRM2* at centro‐parietal regions. The GABAergic and cholinergic systems interact in local inhibitory circuits, and therefore are likely to impact cortical synchronization (i.e., coherence), which animal models suggest may impair behavioral flexibility and contribute to memory deficits.^119^ These early findings are now emerging in much larger GWAS studies of alcohol dependence and related phenotypes. For example, variants in *GABRA2*, *CHRM2 and GRM8* have been implicated in large GWAS (>1 million participants) of alcohol consumption, risk‐taking behaviors, and related addictive phenotypes^120^, ^121^, ^122^ (e.g., smoking). Variants in *GABRA2* have also been associated with EEG‐based phenotypes,^123^ but in relatively smaller samples (>10 thousand participants) given the uniqueness of these datasets.

#### 3.1.2 Genome‐wide association studies (GWAS) of neurophysiological phenotypes

Genetic data from COGA's enriched and ancestrally diverse families have been used independently as well as in consortia efforts (e.g., ENIGMA‐EEG) to conduct GWAS of the EEG‐based phenotypes described in Table 2. In one example, a family‐based GWAS of frontal theta EROs during P300 to targets in the visual oddball task identified several genome‐wide significant non‐coding variants as well as a synonymous SNP (rs702859) within *KCNJ6* (the gene encoding GIRK2, G protein‐activated inward rectifier potassium channel 2).^42^ Converging data from other research groups has demonstrated that GIRK2 activation contributes to slow inhibitory postsynaptic potentials important in modulating neuronal excitability. GIRK channels are directly activated by ethanol^124^ and play an important role in both ethanol‐ and opioid‐induced analgesia. Low frontal theta ERO activation is associated with AUD^35^ and those at risk,^36^ and these genetic findings suggest that GIRK2 activation accounts for some of the variations in frontal theta oscillations seen in COGA families. We used COGA's longitudinal data to examine the neurodevelopmental trajectories of these frontal theta ERO phenotypes in the same visual oddball task during adolescence and young adulthood^44^, ^52^ and found age‐ and sex‐specific effects of the *KCNJ6* variants^52^ between the aged of 12–25. In another example of this approach, in the same adolescent and young adult sample in a reward processing task, frontal theta power EROs increased as a function of the minor allele dose of *KCNJ6* SNP rs702859 during the loss condition^65^ (Figure S1) These findings have implications for understanding the mechanisms through which genetics can influence neuronal circuits and indirectly, reward related behaviors. Ongoing research aims to determine functional significance of these GIRK2 variants in iPSC studies in COGA families^125^ (see 5. Functional Genomics).

COGA conducted the first neural AUD endophenotype GWAS among individuals of African ancestry (AA) and reported genome‐wide significant association between beta EEG power and chromosome 3 variants that influence the expression of *BCHE* (butyrylcholinesterase^27^). Several of these variants were also associated with AUD in COGA, and the results replicated in the Yale‐Penn Study of Addiction, an independent sample of AA adults.^27^ These variants were also associated with ‘heavy episodic drinking’ among adolescent COGA offspring,^27^ suggesting a role of these loci in neural and behavioral disinhibition across different stages of the lifespan. COGA recently conducted the first EEG interhemispheric coherence GWAS, identifying loci in an intergenic region on chromosome 18 that were associated with resting state EEG theta coherence and had both age and sex‐specific effects. These chromosome 18 variants were also associated with higher number of drinks on one occasion and DSM‐5 AUD symptoms in COGA families and associated with alcohol drinker status and alcohol intake frequency in the UK Biobank, an independent sample.^126^ These findings provide support for the role of genetic variants on chromosome 18q23 in regulating both neural connectivity and alcohol use behaviors, potentially via dysregulated myelination. Interestingly, these variants were also associated with corpus callosum volume in a subset of COGA participants and UK Biobank participants. COGA will continue leading efforts to replicate and expand this work independently and in collaboration with the ENIGMA Consortium‐EEG Workgroup^117^ (11 studies, total N: 17,168).

#### 3.1.3 Polygenic scores (PGS) and neurophysiological phenotypes

The advent of polygenic scores (PGS), note, also described as polygenic risk scores (PRS),^127^, ^128^ which are an aggregate of genetic information from GWAS, permits characterization of the complex interplay of genetic influences on neurodevelopmental trajectories of brain function and risk for AUD. COGA has examined whether polygenic score for DSM‐IV AD (AD PGS)^129^ was associated with developmental trajectories of interhemispheric and intrahemispheric neural connectivity from adolescence to young adulthood (aged 12–32). AD PGS was found to affect development of frontal‐central alpha connectivity in young adult males, but not females,^130^ and was also associated with decreased planning and problem solving skills and poorer visuospatial working memory (Figure S2).^131^ In another recent study, COGA researchers examined the associations between P3 amplitude, PGS for behavioral dyscontrol (EXT PGS), and self‐report of externalizing behaviors.^122^, ^132^ Investigators examined these associations among adolescents (12–17) and young adults (18–32) of both European and African ancestry. Both the EXT PGS and P3 amplitude were associated with externalizing behaviors, but the EXT PGS was not significantly associated with P3 amplitude. The results suggest that genetic liability for behavioral dyscontrol and P3 amplitude are each uniquely contributing to the expression of externalizing behavior, perhaps indexing different facets of externalizing risk.^132^

Across linkage studies, GWAS, and PGS, COGA is uniquely positioned to address multiple facets of complex clinical phenomena, with expertise in translation of findings between brain function, genomics and substance use. The consortia continues to work collaboratively internally and externally to enhance our understanding of mechanisms underlying AUD and related disorders, including in understudied ancestrally diverse populations.

### 3.2 How does the social–environmental context (and interaction with genetic risk factors) impact brain functioning and ultimately impact risk for AUD?

Social–environmental experiences throughout the life course, such as interpersonal influences (parenting, peers, romantic relationships) and traumatic stress, may alter neurophysiological and behavioral development, thereby increasing risk for AUD and related psychopathology over and above the contributions of genetics and neurophysiology.^133^, ^134^ COGA has examined the influence of social‐environmental context on neurocognitive function and AUD as well as how genetic risk may moderate these associations. For example, COGA examined the association of childhood traumatic experiences with developmental trajectories of brain function during response inhibition.^66^ Data were drawn from the COGA prospective cohort, comprising offspring from high‐risk and comparison families who were aged 12–22 at enrollment, with follow ups at two‐year intervals since 2004 (see 2. Sample and Clinical Data). Individuals exposed to sexual assaultive trauma prior to age 10 had slower rates of change in developmental trajectories of frontal theta during response inhibition. Importantly, these effects remained significant after accounting for other traumatic exposures, parental history of AUD and participants' substance use, but not measures of impulsivity. Lagged frontal neurophysiological development during response inhibition may reflect delays in frontal lobe development, synaptic pruning and/or cortical maturation involving neural circuits. These same areas were associated with increased risk for internalizing psychopathology and symptoms of AUD in young adulthood.^66^ These findings support the hypothesis that changes in neurocognitive development related to early sexual trauma exposure may increase risk for mental health and substance use problems in young adulthood. Building on this work, COGA^67^ observed that female, but not male, participants who experienced a sexual assaultive trauma, and were from families more densely affected with AUD, had higher rates of PTSD symptoms. In addition, exposure to nonsexual assaultive trauma was associated with poorer neurocognitive performance (planning and problem‐solving skills on the Tower of London test) in young adulthood. Parenting has been found to impact offspring brain development, neurocognitive function, risk and resilience for AUD via both genetic and socio‐environmental factors. Using data from the COGA prospective cohort, COGA demonstrated that greater ‘closeness with father’ was associated with larger P300 amplitude and higher frontal theta power in offspring.^33^ Also, ‘closeness with mother’ was associated with less binge drinking in offspring. Importantly, these associations remained significant beyond other relevant risk factors such as parental AUD, other substance use, income, education and offspring characteristics such as impulsivity. These findings suggest that close relationships with parents, during the critical period of adolescence, may mitigate the “neurodevelopmental lag” in individuals with heightened vulnerability to AUD and may contribute to more efficient neurocognitive functioning.^33^

### 3.3 How do these multi‐modal risk and protective factors fit together to influence the development and course of AUD?

Taking advantage of the wealth of our multimodal data and our interdisciplinary expertise, COGA has used machine learning (ML) methods as one approach to understand the interplay of complex factors involved in risk and resilience to as well as recovery from AUD. ML methods take an atheoretical approach to aggregating high‐dimensional data.^135^ COGA has used ML algorithms to build models with the goal of identifying individuals at higher risk of developing AUD. For example, we reported that ML models combining EEG and AUD associated genetic variants outperformed other models based on a single type of data, suggesting that each contributed unique and significant information.^136^ Using a Random Forests method, we found that EEG hyperconnectivity across the default mode network regions, PGS for AUD, alcohol consumption and related health consequences, elevated neuroticism, increased harm avoidance, and fewer positive life events could all be used to classify individuals who would develop alcohol induced memory problems 20 years later.^31^ More recently, COGA has used ML algorithms to predict the difference in AUD recovery status, identifying several discriminative features, including PGS related to alcohol use, personality and psychopathology, psychosocial factors and electrophysiological indicators including lower default mode network and fusiform connectivity and higher insula connectivity^137^ (Figure S3). Taken together, these ML findings highlight the strength of the multidomain data and analytical expertise in COGA and provide examples of how this information can be synthesized across modalities to identify risk factors and improve prediction of the development of AUD, and patterns of persistence, resistance and recovery from AUD, across the lifespan.

## 4 CONCLUSIONS AND FUTURE DIRECTIONS

Alcohol use disorder and related health conditions result from a complex interaction of genetic, neural and environmental factors, with differential impacts across the lifespan. From its inception, COGA has focused on the importance of brain function as it relates to the risk and consequences of alcohol use and AUD, through the examination of noninvasively recorded brain electrical activity and neuropsychological tests. COGA's sophisticated neurophysiological and neuropsychological measures, together with other rich multi‐modal family and longitudinal data, have allowed us to disentangle brain‐related risk and resilience factors from the consequences of prolonged and heavy alcohol use in the context of genomic and social‐environmental influences over the lifespan. COGA has led the field in identifying genetic variation associated with brain functioning, which has advanced the understanding of how genomic risk affects AUD. To date, COGA has amassed an impressive collection of neurophysiological and neuropsychological data on close to 10,000 participants (Table 1) who have also been carefully assessed with diagnostic interviews, behavioral questionnaires, and for whom DNA samples have been collected—all of which are available to the research community. As the complexity of genetic analyses has grown, so too have the tools used in the investigation of brain function. Building upon the past and looking towards the future, COGA is in a unique position to continue to contribute to the field by further investigating the neurophysiological and neuropsychological brain function data and its critical role in linking genetic influences on neural systems to the development and course of AUD and related disorders.

(B) Bipolar electrode pairs used as the coherence measures of resting state EEG.

## Supporting information


**Data S1.** Supporting Information

## Data Availability

COGA data are available in dbGaP (phs000125, phs000763, phs000976, phs001208), or via an application to the National Institute on Alcohol Abuse and Alcoholism (https://www.niaaa.nih.gov/research/major‐initiatives/collaborative‐studies‐genetics‐alcoholism‐coga‐study), or a COGA investigator sponsored secondary analysis proposal.
