# Supplementary material for: The collaborative study on the genetics of alcoholism: Brain function
Source: Genes Brain Behav. 2023 Aug 17;22(5):e12862. doi: 10.1111/gbb.12862 (PMC10550791; doi:10.1111/gbb.12862)
Supplement: Supplementary file 1 — Data S1. Supporting Information [file GBB-22-e12862-s001.docx]

**Supplemental Material**

# COGA Neurophysiological Protocols, Measures and Findings

# EEG Recording Procedures

EEG recording procedures have changed somewhat over the course of the past 30 years of data collection. Here we report on the current (as of 2023) procedures^1,2^ but note where there have been changes and the previous standards^3,4^. Participants are seated in a dimly lit sound-attenuated chamber, fitted with an electrode cap (Electro-Cap International, Inc.) with 64 electrodes (extended 10-20 system)^5,6^, with nose as the common reference and forehead as the ground location (**Fig 1**). Eye movements and blinks are recorded with monopolar electrodes placed on bilateral canthi of the eyes and above the left eye as well as bipolar electrodes placed above and below left eye orbitals^7^. Electrode impedances are maintained below 5K ohms. EEG is amplified by a factor of 10,000 by Neuroscan amplifiers; the resulting signal is fed into an analog to digital converter on the recording computer with 32-bit output (initially with 16-bit output) filtered between DC and 100 Hz and sampled at 500 Hz (initially at 256 Hz). Response inputs are also recorded on this computer. For task-related protocols, stimuli are presented on a monitor in the chamber whose output is controlled by a second computer. Software for the x86 recording and control computers is provided by the Neuroscan system (Version 4.1-4.3) (Compumedics, Inc., El Paso, TX) with different sampling rates at different phases (i.e., 256, 512, 500 Hz). At the start of the COGA, the COGA software system (an in-house effort) running on Masscomp computers was used for a single recording and control computer. Initially, recordings were made with either 21 or 32 electrodes scalp electrodes; subsequently, 64 electrodes were used. The signals were amplified with a gain of 10,000 by a set of amplifiers (Sensorium, Charlotte, VT) with bandpass of 0.02–100 Hz.

# EEG/ERP/ERO Processing

EEG data is subjected to quality control checking, which involves correcting for known artifacts (movement, EMG, eye movement, DC shifts, incorrect or missing responses, etc.) before systemic analysis. In most cases, data sampled at a frequency greater than 256 Hz is downsampled to that frequency in order to be consistent with earlier data sampling at 256 Hz.

For resting-state EEG analyses, we employ two different methods: **1)** **Signal processing analysis** of the recorded scalp data, in which bipolar derivations are used to reduce volume conduction effects^2,8,9^. Fourier transform and other methods are used to compute power and interhemispheric and intra-hemispheric coherence in single frequency and standard EEG bands. Synchrony measures are computed to describe the relationship between two or more signals. **2) Source localization** methods attempt to identify the origin of the recorded scalp activity in different parts of the brain. We use LORETA methods to measure current density^10,11^ and apply other inverse solutions using publicly available toolboxes (e.g., eLORETA, MNE) to compute EEG source functional connectivity^12,13^ on resting EEG data.

To derive ERPs, maxima, minima, and latencies of key ERP components are determined using a semi-automated in-house method. For EROs, continuous wavelet transform^14^ and S-transform^1,15,16^ methods are used to extract power and synchrony time-frequency measures of task-related activity. Power measures are extracted from individual trials and averages of all trials for each task condition, providing measures of total and evoked activity in time-frequency regions of interest^15^.

# COGA Neurophysiological Tasks and Measures

| **Table S1. COGA Neurophysiological, Neurocognitive and Neuroaffective Protocols and Measures** | | |
| --- | --- | --- |
| **Task Name [Cognitive Components]** | **Stimuli and Task Description** | **Measures** |
| ***_a,b,c,d_***  **Resting State EEG (eyes closed (EEC) & eyes open (EEO))^4^**  [Resting state brain activity, (e.g., Default Mode Network (DMN))] | EEG recorded continuously for 4.25 minutes at 500 Hz sampling rate in two states:  (a) resting while eyes are closed (EEC)  (b) resting with eyes open (EEO) | Absolute/Relative power: Delta (1-3.5 Hz); Theta (3.5-7.5 Hz); Alpha (8-12.5 Hz); Beta (13-28 Hz); Gamma (28-45 Hz); Theta/beta ratio; Inter/intra-hemispheric coherence; functional lagged connectivity-eLORETA |
| ***_a,b,c,d_***  **Visual Oddball (VP3)^1,15^**  [Stimulus discrimination; attention, template matching; working memory; decision making] | 210 squares (non-targets), 35 X’s (targets) and 35 colored geometric polygons (novels) with ISI of 1600 ms. Participant presses button only to rare X’s. | ERP and ERO measures to Target stimuli: N100, N200, & P300 amplitude/latency; ERO: evoked/total power: Delta, Theta, Alpha, Beta, & Gamma. |
| ***_a,b,c,d_***  **Auditory Oddball (AO)^17-19^**  [Stimulus discrimination; attention, template matching; working memory; decision making] | 50 rare high tones (1600 Hz), and 350 frequent low tones (600 Hz) with ISI of 1500 ms at 70 db. Participant presses button only to rare high tones. | ERP and ERO measures to Target Stimuli: N100, N200 and P300; Evoked and Total power in Delta, Theta, Alpha and Gamma bands. |
| ***_a,b,c,d_***  **Semantic Priming (ANT)^20,21^**  [Semantic processing; context differentiation] | 150 non-words (jumbles), 50 primes, 50 antonyms, and 50 unrelated words. Antonyms directly follow the primes. The ISI is 1600 ms. Participant presses button #1 to word and button #4 to non-word | ERP and ERO measures to Primed and Unprimed words: P300 and N400 amplitude & latency; Evoked and Total power of Theta, Delta and Gamma bands. |
| ***_b,c_***  **Go/NoGo task (GNG)^22,23^**  [Response Inhibition/Conflict monitoring] | Participant presses button to 50 Go (triangles pointing up or down) and not to 50 NoGo (triangles pointing left or right) for 100 ms. Response feedback stimuli “$” (correct) or “X” (wrong) for 200 ms. ITI is 2400 ms. Speed and accuracy stressed. | ERP and ERO measures to Go and NoGo (response inhibition) conditions: N200, P300 amplitude/latency; ERO: evoked/total power: Delta, Theta, Alpha, Beta, & Gamma. Errors of omission, commission, RT. |
| ***_b,c_***  **Monetary Gambling Task (MGT)^16,24,25^**  [Reward/outcome evaluation; Decision making; Performance monitoring.] | 172 choice stimuli, either “10” or “50”; participant bets one of the amounts; outcome stimulus gives feedback of loss (red) or gain (green) for bet. Stimulus duration = 800 ms and ISI = 1500 ms. | ERP and ERO measures to feedback from Loss and Gain conditions: N200, P300 amplitude/latency; ERO: evoked/total power/synchrony: Delta, Theta, Alpha, Beta, Gamm |
| ***_b,c_***  **CATs Tower of London Test (TOLT)**^26^  [Planning & problem-solving ability; Speed of planning] | Move colored beads on 3-5 pegs until achieving the specified goal position in as few moves as possible. | Planning and Problem-solving ability:  Accuracy; Excess moves made; Correct trials within minimum moves; Average pick-up time, Average total time. |
| ***_b,c_***  **CATs Visual Span Test (VST)**^26^  [Visuospatial attention span; Visual working memory] | Forward and backward span  1) Repeat increasingly complex sequences of flashing squares (2-8) by clicking on squares. 2) Reverse order. | Visualspatial memory span and working memory: Highest sequence is achieved at each level. |
| ***_c,d_***  **NIH Toolbox Cognitive Battery**^27^  [Attention, episodic and working memory, language/intelligence, executive functions, and processing speed] | *Subtests:* Flanker Inhibitory Control and Attention Test, Picture Sequence Memory Test, List Sorting Working Memory Test, Picture Vocabulary Test, Oral Reading Recognition Test, Flanker Inhibitory Control and Attention Test, Dimensional Change Card Sort Test, Pattern Comparison Processing Speed Test. | Composite fluid cognitive scores, Composite crystallized cognitive scores, Composite cognitive scores |
| ***_c,d_***  **NIH Toolbox Emotion Battery (NIHT-EB)**^28^  [Emotional functioning] | *Domains:* Negative Affect, Psychological Well-Being, Stress and Self-Efficacy, and Social Relationships. | Emotional functioning. |
| **_a_ Initial COGA Sample (1989-2004)**  **_b_** **Prospective Study Sample:** Multiple assessments every 2 years during adolescence and young adulthood from 2004-2019. Added Frontal Lobe Battery in Prospective study to assess aspects of frontal lobe in development during that period. Phenotypes derived from this data can be used for studies of neurodevelopmental trajectories.  **_c_** **Lifespan Project: Midlife (ML):** Prospective Study sample as they enter mid-life (33-49);  **_d_** **Lifespan Project Latelife (LL)**: Initial sample now aged >50 (One or two previous assessments ~ 20 years ago) | | |

# Supplemental Figures

A detailed summary of these and other electrophysiological findings in COGA are reported in the main manuscript.

^
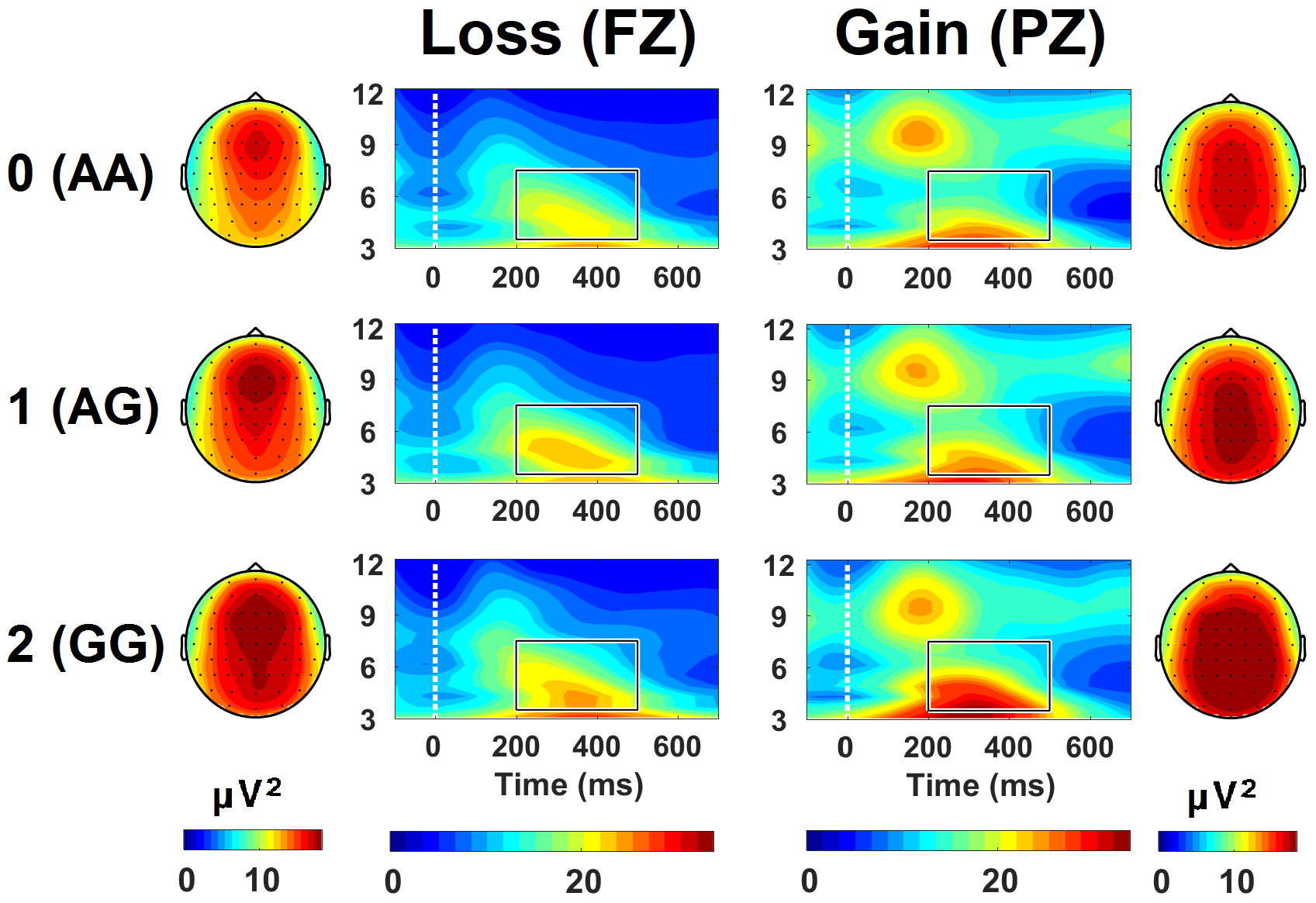
^

**Figure S1**. Theta power (in μV2) across *KCNJ6* genotypes for synonymous SNP (rs702859) during responses to loss (at FZ electrode) and gain (at PZ electrode) (Note Loss response has an anterior focus, while Gain has a posterior focus). Time-Frequency (TF) plots (*x*-axis with time in ms relative to stimulus onset and *y*-axis with the frequency of EEG oscillations in Hz; the amount of activity in each TF cell is color-coded). Topographic maps show the scalp distribution of the total amount of activity in the region of interest in the TF space. The dotted vertical line (at 0 ms) in the TF plots represents the onset of the outcome stimulus. The smaller rectangles within the time-frequency plots represent the theta power (3.5–7.5 Hz within 200–500 ms) post-outcome stimulus. During the evaluation of loss as well as gain, there is an additive effect of genotypes [GG > AG > AA] with increasing power corresponding to the number of the minor allele(s) [Kamarajan et al (2017)^25^].


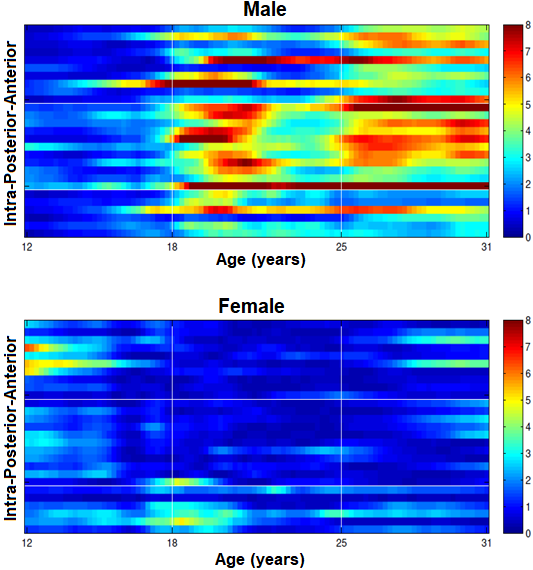


**Figure S2:** Association (–log10 p-value) of DSM-IV Alcohol Dependence PRS (p<0.001 threshold) at coherence pairs (y-axis) organized (top to bottom) from interhemispheric anterior pairs to posterior pairs, and intra-hemispheric pairs (Fig 1b); prominent associations with fronto-central, tempo-parietal, centro-parietal, and parietal-occipital interhemispheric high alpha coherence are observed only among males from ages 18-31 [Meyers et al (2019)^9^].

^
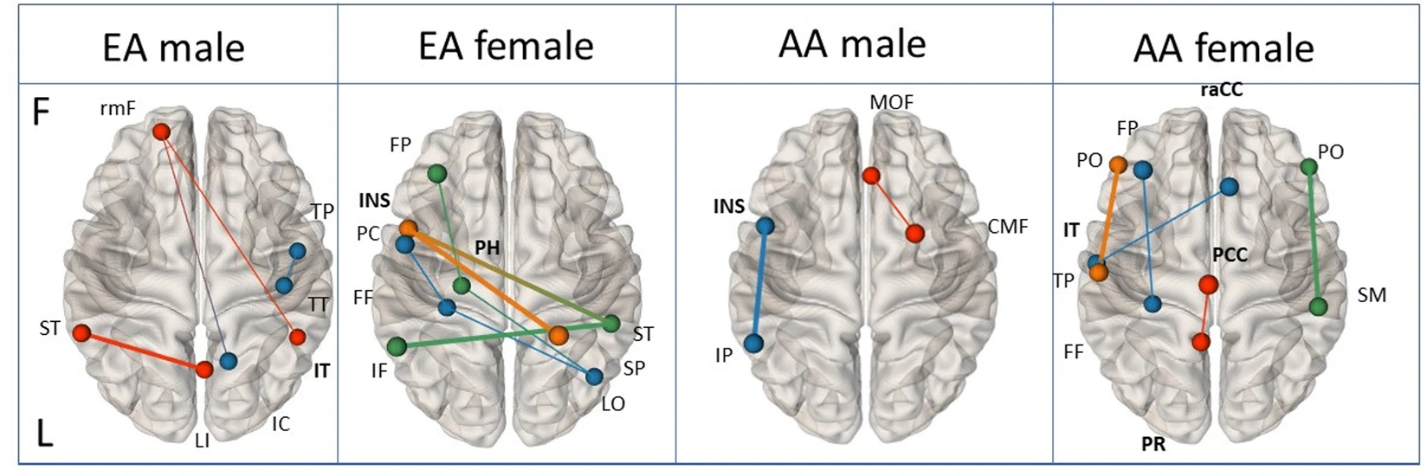
^

**Figure S3: EEG functional connectivity AUD remission biomarkers.** AUD remission prediction models reveal ancestry/sex group-specific brain connectivity biomarkers discriminating between those who recovered from AUD to those who did not. Results highlight lower connectivity in theta (blue) and gamma (red) in areas related to DMN (in bold -IT, PCC, PR, raCC, PH) and higher connectivity in theta (blue) and beta (orange) between insula and inferior and superior parietal regions respectively (in bold INS, IP, IT) specific to every sex and ancestry predicting the maintenance of AUD. blue-theta, green-alpha, orange-beta, red-gamma. Thinner lines—lower connectivity, Thicker lines—higher connectivity. CMF Caudal middle frontal, FF fusiform, FP frontal pole, INS insula, IC Isthmus cingulate, IP inferior parietal, IT inferior temporal, LI lingual, LO Lateral Occipital, MOF medial orbitofrontal, PC paracentral, PCC posterior cingulate cortex, PH parahippocampus, PO pars orbitalis, PR precuneus, raCC rostral anterior cingulate cortex, SM supramarginal, rmF Rostral middle frontal, TP temporal pole, TT Transverse temporal, SP Superior parietal, ST Superior temporal [Kinreich et al (2021)^12^].

| **Textbox S1: Glossary of terminology related to brain electrophysiology** |
| --- |
| **Electrode:** The *electrode* is the sensor placed on the scalp to measure EEG.  **Montage:** The *montage* represents a conventional arrangement of electrodes on the scalp (Fig 1).  **Artifact:** A physiological potential difference due to (a) other electrophysiological signals such as those related to eye blinks and movements (electrooculogram, EOG), electrocardiogram (ECG), or muscle activity (electromyogram, EMG), or (b) extracerebral factors, such as instrumental distortion or malfunction, movement of the patient, or ambient electrical noise.  **Filtering:** an EEG analysis method for isolating EEG oscillations in the frequency range of interest by removing oscillations at other frequencies, typically below 0.1 or 1 Hz or above 40 or 50 Hz Hz).  **EEG:** Electroencephalogram (EEG) measures the electrical rhythmic activity (voltage oscillations) of the brain recorded non-invasively by electrodes placed on the scalp, comprising a wide range of spectral frequencies:  **Spectral frequencies**: number of wave cycles or peaks that occur in an EEG signal per second and is measured in hertz (Hz). These are subdivided into EEG bands as follows:   - **Delta band:** Frequency band of 0.1–3.5 Hz (wave cycles per second). Greek letter: δ. - **Theta band:** Frequency band from 3,5−7.5 Hz. Greek letter: θ. - **Alpha band:** Frequency band of 7.5–12.5 Hz (wave cycles per second). Greek letter: α. - **Beta band:** Frequency band of 12.5–28 Hz (wave cycles per second). Greek letter: β. - **Gamma band**: Frequency band from >28 to 80 Hz (wave cycles per second). Greek letter: γ.   **Spectral Power:** Amount of energy within a frequency band of a given EEG signal.  **ERP:** Event-related potentials (ERP) are time-locked brain responses to a specific sensory, cognitive, or behavioral event that are averaged across trials and are quantified in terms of consecutive scalp potential deflections (ERP components, or waves) conventionally labeled according to their polarity (P for positive, N for negative) and peak latency in milliseconds relative to the event onset (e.g., P100, N100).   - **P300 or P3:** is a large positive ERP component that occurs between 250 to 700 ms after a “target” stimulus in a series of standard stimuli, usually in an “oddball” task, with maximal amplitude (brain activation) over the mid-parietal region of the scalp. It is not related to the physical features of the stimulus but a person’s reaction to it. - **N2 or N200:** is a negative-going ERP component that peaks 200-350ms after stimulus onset and is usually maximal over anterior scalp sites, with executive cognitive control functions (e.g., NoGo (response inhibition) condition in a Go/NoGo task). - **N1 or N100:** is a large negative-going ERP component that peaks between 80 and 120 milliseconds after the onset of a stimulus and is distributed mostly over the frontal-central region of the scalp for auditory tasks and more posterior for visual tasks. - **N4 or N400:** is a negative-going ERP component that peaks around 400 milliseconds after meaningful words or symbols and is typically maximal over central-parietal electrode sites. - **Amplitude:** In relation to ERP components, amplitude refers to the strength or intensity of the neural activity measured in microvolts of electrical energy. Peak amplitude is measured relative to either the baseline EEG signal preceding the onset of the event or the peak value of the preceding ERP component (i.e., peak-to-peak). - **Latency:** The time interval between the onset of an event (stimulus or motor response) and the point of maximal value (peak) of a defined component, usually represented in milliseconds.   **ERO:** Event-related oscillations are time-specific changes in the power of EEG oscillations in different frequency bands during a cognitive task.  **Functional Connectivity:** refers to the statistical relationship between specific physiological signals in time (e.g., EEG, MEG, and fMRI). It can be derived using many different methods, including coherence, phase synchronization, phase-slope index, and Granger causality.  **Coherence:** The mathematical measure of the degree of similarity (phase consistency) of EEG oscillations recorded at two sensors. Coherence values are frequency-specific and range from 0 to 1.  **Current (Source) Density:** Estimated EEG activity derived from cortical sources of neural activity for a given scalp recorded EEG trace. This can be obtained in either 2-dimensional surface data or 3-dimensional volumetric data.  **LORETA/sLORETA/eLORETA:** A set of linear inverse solution methods that can reconstruct cortical sources of electrical activity from the scalp EEG data using current density computations.  **Default Mode Network (DMN):** A resting-state brain network primarily composed of the dorsal medial prefrontal cortex, posterior cingulate cortex/precuneus, and angular gyrus, which reflects brain activity when a person at wakeful rest, and possibly involved in thinking about others, thinking about themselves, remembering the past, and planning for the future. |

**References**

1. Rangaswamy M, Jones KA, Porjesz B, et al. Delta and theta oscillations as risk markers in adolescent offspring of alcoholics. *Int J Psychophysiol.* 2007;63(1):3-15. doi:10.1016/j.ijpsycho.2006.10.003

2. Meyers JL, Zhang J, Chorlian DB, et al. A genome-wide association study of interhemispheric theta EEG coherence: implications for neural connectivity and alcohol use behavior. *Mol Psychiatry.* 2021;26(9):5040-5052. doi:10.1038/s41380-020-0777-6

3. Porjesz B, Begleiter H. Event-related potentials in COA's. *Alcohol Health Res World.* 1997;21(3):236-240

4. Rangaswamy M, Porjesz B, Chorlian DB, et al. Beta power in the EEG of alcoholics. *Biol Psychiatry.* 2002;52(8):831-842. doi:10.1016/s0006-3223(02)01362-8

5. Chatrian GE, Lettich E, Nelson PL. Ten percent electrode system for topographic studies of spontaneous and evoked EEG activities. *Am J EEG Technol.* 1985;25(2):83-92

6. Chatrian GE, Lettich E, Nelson PL. Modified nomenclature for the "10%" electrode system. *J Clin Neurophysiol.* 1988;5(2):183-186

7. Croft RJ, Barry RJ. EOG correction: comparing different calibration methods, and determining the number of epochs required in a calibration average. *Clin Neurophysiol.* 2000;111(3):440-443. doi:10.1016/s1388-2457(99)00256-4

8. Chorlian DB, Rangaswamy M, Porjesz B. EEG coherence: topography and frequency structure. *Exp Brain Res.* 2009;198(1):59-83. doi:10.1007/s00221-009-1936-9

9. Meyers JL, Chorlian DB, Johnson EC, et al. Association of Polygenic Liability for Alcohol Dependence and EEG Connectivity in Adolescence and Young Adulthood. *Brain Sci.* 2019;9(10):280. doi:10.3390/brainsci9100280

10. Kamarajan C, Rangaswamy M, Tang Y, et al. Dysfunctional reward processing in male alcoholics: an ERP study during a gambling task. *J Psychiatr Res.* 2010;44(9):576-590. doi:10.1016/j.jpsychires.2009.11.019

11. Pandey AK, Kamarajan C, Tang Y, et al. Neurocognitive deficits in male alcoholics: an ERP/sLORETA analysis of the N2 component in an equal probability Go/NoGo task. *Biol Psychol.* 2012;89(1):170-182. doi:10.1016/j.biopsycho.2011.10.009

12. Kinreich S, McCutcheon VV, Aliev F, et al. Predicting alcohol use disorder remission: a longitudinal multimodal multi-featured machine learning approach. *Transl Psychiatry.* 2021;11(1):166. doi:10.1038/s41398-021-01281-2

13. Kamarajan C, Pandey AK, Chorlian DB, et al. Predicting alcohol-related memory problems in older adults: A machine learning study with multi-domain features. *bioRxiv.* 2023:2022.2012.2030.522330. doi:10.1101/2022.12.30.522330

14. Chorlian DB, Porjesz B, Begleiter H. Determination of human EEG alpha entrainment ERD/ERS using the continuous complex wavelet transform. *Independent Component Analyses, Wavelets, and Neural Networks.* 2003;5102:145-155. doi:10.1117/12.485937

15. Jones KA, Porjesz B, Chorlian D, et al. S-transform time-frequency analysis of P300 reveals deficits in individuals diagnosed with alcoholism. *Clin Neurophysiol.* 2006;117(10):2128-2143. doi:10.1016/j.clinph.2006.02.028

16. Kamarajan C, Pandey AK, Chorlian DB, et al. Deficient Event-Related Theta Oscillations in Individuals at Risk for Alcoholism: A Study of Reward Processing and Impulsivity Features. *PLOS ONE.* 2015;10(11):e0142659. doi:10.1371/journal.pone.0142659

17. Alexander JE, Polich J, Bloom FE, et al. P300 from an auditory oddball task: inter-laboratory consistency. *Int J Psychophysiol.* 1994;17(1):35-46. doi:10.1016/0167-8760(94)90053-1

18. Ramachandran G, Porjesz B, Begleiter H, Litke A. A simple auditory oddball task in young adult males at high risk for alcoholism. *Alcohol Clin Exp Res.* 1996;20(1):9-15. doi:10.1111/j.1530-0277.1996.tb01035.x

19. Chorlian DB, Rangaswamy M, Manz N, et al. Genetic correlates of the development of theta event related oscillations in adolescents and young adults. *Int J Psychophysiol.* 2017;115:24-39. doi:10.1016/j.ijpsycho.2016.11.007

20. Roopesh BN, Rangaswamy M, Kamarajan C, et al. Priming deficiency in male subjects at risk for alcoholism: the N4 during a lexical decision task. *Alcohol Clin Exp Res.* 2009;33(12):2027-2036. doi:10.1111/j.1530-0277.2009.01042.x

21. Roopesh BN, Rangaswamy M, Kamarajan C, Chorlian DB, Pandey AK, Porjesz B. Reduced resource optimization in male alcoholics: N400 in a lexical decision paradigm. *Alcohol Clin Exp Res.* 2010;34(11):1905-1914. doi:10.1111/j.1530-0277.2010.01279.x

22. Kamarajan C, Porjesz B, Jones K, et al. Event-related oscillations in offspring of alcoholics: neurocognitive disinhibition as a risk for alcoholism. *Biol Psychiatry.* 2006;59(7):625-634. doi:10.1016/j.biopsych.2005.08.017

23. Pandey AK, Kamarajan C, Manz N, Chorlian DB, Stimus A, Porjesz B. Delta, theta, and alpha event-related oscillations in alcoholics during Go/NoGo task: Neurocognitive deficits in execution, inhibition, and attention processing. *Prog Neuropsychopharmacol Biol Psychiatry.* 2016;65:158-171. doi:10.1016/j.pnpbp.2015.10.002

24. Kamarajan C, Pandey AK, Chorlian DB, et al. Reward processing deficits and impulsivity in high-risk offspring of alcoholics: A study of event-related potentials during a monetary gambling task. *Int J Psychophysiol.* 2015;98(2 Pt 1):182-200. doi:10.1016/j.ijpsycho.2015.09.005

25. Kamarajan C, Pandey AK, Chorlian DB, et al. A KCNJ6 gene polymorphism modulates theta oscillations during reward processing. *Int J Psychophysiol.* 2017;115:13-23. doi:10.1016/j.ijpsycho.2016.12.007

26. Davis HP, Keller F. *Colorado Assessment Tests (CATs), Version 1.2.* Colorado Springs, Colorado2002.

27. Weintraub S, Dikmen SS, Heaton RK, et al. Cognition assessment using the NIH Toolbox. *Neurology.* 2013;80(11 Suppl 3):S54-64. doi:10.1212/WNL.0b013e3182872ded

28. Babakhanyan I, McKenna BS, Casaletto KB, Nowinski CJ, Heaton RK. National Institutes of Health Toolbox Emotion Battery for English- and Spanish-speaking adults: normative data and factor-based summary scores. *Patient Relat Outcome Meas.* 2018;9:115-127. doi:10.2147/PROM.S151658
